# Human resources for emergency obstetric care in northern Tanzania: distribution of quantity or quality?

**DOI:** 10.1186/1478-4491-3-5

**Published:** 2005-07-29

**Authors:** Øystein Evjen Olsen, Sidney Ndeki, Ole Frithjof Norheim

**Affiliations:** 1 Center for International Health, University of Bergen, Norway. For correspondence: DBL – Institute for Health Research and Development and Primary Health Care Institute, Iringa, Tanzania; P.Box 105297, Dar Es Salaam, Tanzania; 2 Center for Educational Development and Health (CEDHA), Arusha, Tanzania; 3 Section for Medical Ethics and Philosophy of Science, Department of Public Health and Primary Care, University of Bergen, Norway, and Center for International Health, University of Bergen, Norway

## Abstract

**Background:**

Health care agencies report that the major limiting factor for implementing effective health policies and reforms worldwide is a lack of qualified human resources. Although many agencies have adopted policy development and clinical practice guidelines, the human resources necessary to carry out these policies towards actual reform are not yet in place.

**Objectives:**

The goal of this article is to evaluate the current status of human resources quality, availability and distribution in Northern Tanzania in order to provide emergency obstetric care services to specific districts in this area. The article also discusses the usefulness of distribution indicators for describing equity in the decision-making process.

**Methods:**

We conducted a quantitative facility survey in six districts of Northern Tanzania. We collected data from all 129 facilities that provide delivery services in the study area. The data includes information on the emergency obstetric care indicators, as described by the WHO/UNICEF/UFPA guidelines for monitoring the provision of obstetric care. The inventory also includes information on the numbers of qualified health personnel at the basic and comprehensive emergency obstetric care level. We analysed the distribution and workload of the available human resources in a wider policy context with a particular focus on equity, use and quality, by means of descriptive statistics and the Spearman's correlation test.

**Results:**

We determined that there are adequate human resources allocated for health care provision in Tanzania, according to national standards. Compared to similar countries however, Tanzania has a very low availability of health care staff. Most qualified staff are concentrated in a few centralized locations, while those remaining are inequitably and inefficiently distributed in rural areas and lower-level services. Rural districts have restricted access to government-run health care, because these facilities are understaffed. In fact, voluntary agency facilities in these districts have more staff than the government facilities. There is a statistical correlation between availability of qualified human resources and use of services, but the availability of qualified human resources does not automatically translate into higher availability of qualified emergency obstetric care services.

**Conclusion:**

National guidelines for human resources for health care in Tanzania require focused revisions in order to reflect the quality indicators more adequately when monitoring and setting criteria for HR distribution. Availability of qualified personnel as well as institutional management and capacity determine the quality of emergency obstetric care services and personnel. The current wide distribution of staff of inadequate quality should be reconsidered. The use of distribution indicators alone is not useful to properly monitor equity. This article suggests increasing access to high-quality health care instead of distributing low-quality services widely.

## Background and introduction

Simms and co-workers [1] predict that the health care system infrastructure in developing countries such as in Africa will collapse in the very near future. Recent attention has focused on the capacity of health care systems to provide adequate and timely services [1]. The lack of qualified human resources for health care is a major limiting factor in implementing health policies and health reforms in the developing world [2]. One of the major challenges is securing the availability and effective use of qualified human resources (HR). Thus, it is important for health care research to provide those who make and implement health care decisions with up-to-date information regarding the status of current resource allocation, shortcomings and planning strategies.

As a tracer policy, this paper attempts to identify the HR available to implement reformed reproductive health policy in Tanzania. We have chosen to identify personnel who are able to implement policies for reducing maternal mortality by focusing on emergency obstetric care (EmOC) services.

De Brouwere and co-workers [3] point out that maternal mortality in itself is not a good indicator for assessing maternal health care programmes and maternal health. Rather, it is important to assess the unmet obstetric needs and demonstrate the relative importance of adequate health care provision. Therefore, monitoring maternal health now tends to use process measures rather than impact measures as an accepted proxy [4].

Bertrand and Tsui [4], Nirupam and Yuster [5] and WHO are among many researchers and organizations providing comprehensive efforts to select useful process indicators to evaluate reproductive health programs. This article is based on the framework proposed by UNICEF, WHO and UNFPA [6]. This framework asks six basic questions:

1. Are there enough facilities providing emergency obstetric services (EOC)?

2. Are they well distributed?

3. Are enough women using these facilities?

4. Are the right women (those with obstetric complications) using these facilities?

5. Are sufficient quantities of critical services being provided?

6. Is the quality of the services adequate?

The questions in this framework are used to describe equity of access to quality care in terms of availability of EmOC units, distribution of EmOC services and use and quality of these services. These guidelines also provide a tool for defining and monitoring the emergency obstetric care units

We and other researchers as well as government agencies are using the United Nations guidelines more and more to evaluate the availability of obstetric care of good quality. [7-15].

Securing human resources for maternal health services is a key component in achieving the Millennium Development Goals (MDGs) by 2015. Specifically, many governments and organizations recognize that HR have not been targeted sufficiently for previous global development initiatives such as the Sector Wide Approaches and Poverty Reduction Strategy programmes. This trend is changing, and important initiatives are being established to attract policy-makers to this challenge. Examples are the Africa Working Group on Human Resources (part of the Joint Learning Initiative) and the Health Systems Trust focus on HR in southern Africa. There is also a working group in Tanzania comprising representatives from different ministries and partnerships. Results from this working group are not yet available.

It is vital that policy-makers have access to evidence on key aspects of HR management. Diallo and co-workers [16] have provided an overview of potentially important and useful monitoring and evaluation tools. Indicators include the available HR stock, skill mix, working location and equity issues.

The goal of this article is to adapt this framework to determine HR quality, availability and distribution for providing emergency obstetric care services in Northern Tanzania. We analyse the distribution and workload of these available HR in the wider policy context of urban and rural settings, public or private ownership and facility levels in the health care pyramid, with a particular focus on equity, use and quality.

## Methods

### Setting

The study area included two districts in the Kilimanjaro Region and four districts in the Arusha Region (two of which now are in the newly formed Manyara Region) of Tanzania, with a total population of about 1.5 million. We selected these districts to reflect different stages of health sector reform implementation, urban and rural settings and public and private service mixtures. Within these regions we identified and surveyed all facilities (n = 129) providing delivery services at all levels of service (dispensary, health centre, first referral hospital, secondary referral hospital). These included government (G), voluntary agencies (VA) and private, for-profit (PFP) facilities.

### Data collection

The study represented a combination of comprehensive facility survey and document review. We conducted the facility survey through the structured analysis of facility documents (health management information system – (HMIS)) with the aid of a facility manager interview. Additional policy documents were reviewed at the district and regional health authority levels.

We determined the EmOC status of each facility by asking the following questions during the interview with the facility managers:

Were the following services performed at least once during the last 3 months (Yes/No):

1. parenteral antibiotics?

2. parenteral oxytocics?

3. parenteral sedatives/anticonvulsants?

4. manual removal of placenta?

5. removal of retained products?

6. assisted vaginal delivery?

7. blood transfusion?

8. caesarean section?

If the facility had performed functions 1 through 6 listed on the questionnaire, we considered them as basic emergency obstetric care units (BEmOC). If they also performed tasks 7 and 8, we considered them to be comprehensive emergency obstetric care units (CEmOC).

The United Nations guidelines audit established the level of services provided at each facility using the provisional EmOC indicators. These indicators include basic emergency obstetric care (BEmOC) units per 500 000 people, the comprehensive emergency obstetric care (CEmOC) units per 500 000 people, the number of deliveries at the EmOC facilities, the number of complications handled at the EmOC facilities (met need indicator), the caesarean section rates and case fatality rates in the six districts. From facility and district documents we collected a wide range of information including deliveries, complications (both for workload analysis), infrastructure, equipment and staffing resources data.

In addition to listing the total number of employees and their distribution across cadres, the facility managers provided the number of employees capable of conducting procedures at the basic emergency obstetric care and comprehensive emergency obstetric care levels. A pragmatic expectation for this health care provider includes handling a normal delivery with only minor complications (BEmOC capable staff) and handling complicated deliveries, depending upon their ability to perform a caesarean section (CEmOC capable staff).

The study did not attempt to conduct a clinical capability audit of each staff member, but instead relied on the facility manager to impart the number of staff qualified at the time of the survey. Every participant willingly provided the requisite information. Additional information at the district and regional health authority levels included the district health plans, the district processing files (HMIS summary) and the annual regional reports. All activity information pertains to the year 2000. This project employed one competent field assistant in addition to the principal investigator, to help with the data collection process.

### Analysis

We calculated the expected number of births using the population figures from the most recent population census, that of 2002 [17]. From the census we gathered crude birth rates (CBR) of 34.4/1000 in urban districts and 43.5/1000 in rural districts, and growth estimates of 4.0% in the Arusha Region, 1.4% in the Kilimanjaro Region and 3.8% in the Manyara Region [18].

From our own data we compiled and analysed HR availability according to different analytical contexts. These were total HR availability at the district level, availability at each level of health services (dispensary, health centre, first referral hospital and secondary referral hospital) and availability according to ownership of the facilities. The availability of HR in the study area was analysed in terms of workload and output measures by calculating the number of deliveries per qualified worker within the different analytical contexts.

This study uses a complete dataset that includes all facilities providing delivery services and therefore represents a comprehensive overview of all HR for health care in these districts. In this regard, and for the purposes of this article, we conducted descriptive statistical analyses. We used a non-parametric analysis between availability rankings for qualified HR per one unit population (500 000) and United Nations guidelines utilization indicators and quality indicators. The utilization indicators are met need and percentage deliveries in EmOC facilities and the quality indicators are the number of qualified BEmOC and CEmOC facilities per 500 000 people in the six districts (n = 6).

Specifically, we compared direct or inverse correlations between: 1) the availability of qualified HR (BEmOC and CEmOC) and services used by the delivering mothers, and 2) the availability of qualified HR and the quality of the facilities. We used a Spearman's correlation table to find the critical values for the Spearman's ranking coefficient (r_s_) at n = 6.

We used the statistical software package SPSS for Windows, version 11.0.0. Research clearance was obtained from relevant institutions in Tanzania and Norway.

## Results and discussion

In all, our study sample encompassed the six Tanzanian districts and data from all 129 facilities providing delivery services, with 34 756 deliveries, for a quantitative analysis. A summary of the results using the United Nations guidelines process indicators audit is shown in Table 1. Detailed discussions of these results are published elsewhere [14,15].

**Table 1:** Overview of United Nations guidelines process indicators for the survey area

	**Minimum recommended level (United Nations guidelines)**	**Total**	**Moshi urban**	**Hai**	**Arusha urban**	**Arumeru**	**Hanang**	**Mbulu**
BEmOC per 500 000	4	1.6	3.6	0.0	1.9	1.0	2.6	2.3
CEmOC per 500 000	1	4.6	10.7	2.0	11.5	2.1	0.0	4.5
Percentage of facility deliveries in:								
Not EmOC		19.0	4.1	38.6	36.2	14.3	7.6	10.5
BEmOC		2.2	2.7	0.0	4.3	0.2	8.9	0.8
CEmOC	15	34.1	124.1	6.4	112.5	3.7	0.0	42.4
Percentage of total expected complicated deliveries^a^								
EmOC (met need)	100	59.8	319.3	8.2	164.2	8.9	2.1	50.4
Not EmOC	21.4	0.1	30.5	84.1	9.1	2.7		5.8
Caesarean section rate								
At CEmOC	5–15	4.6	23.2	0.9	14.0	0.7	^c^	3.6
Case fatality rate^ab^	1	1.46	1.28	2.09	1.54	1.78	^c^	1.38

^a^Adjusted complications – As described in Plan 3 in the United Nations guidelines

^b^Only at CEmOC facilities recording deaths.

^c^No CEmOC facilities in the district

### Comparing our results with the national standards

Table 2 shows that the aggregated average number of qualified health personnel available at both dispensary and health centre levels is adequate compared to the national standards. Tanzanian HR policy requires that for every 50 000 people there should be five dispensaries and one health centre. For each dispensary there should be two qualified staff and for each health centre, four qualified staff [19]. This means that for a population of 50 000 there should be 14 qualified BEmOC staff, the equivalent of 28 staff per 100 000 people. The HR policies intend for the dispensaries and health centres to be capable of performing BEmOC activities.

**Table 2:** Availability and workload of BEmOC and CEmOC qualified staff at facility level according to public-private mix, levels of services and urban rural contexts

		**Available qualified staff**		**Average qualified staff per facility**			
	**Number of facilities**	**BEmOC**	**CEmOC**	**BEmOC**	**CEmOC**	**Facility deliveries per qualified BEmOC staff (workload)**	**Percentage CEmOC staff of BEmOC staff**

Public-private mix							
Private for-profit	11	50	5	4.5	0.45	6.6	10.0
Voluntary agencies	41	576	36	14.0	0.87	17.1	6.3
Government	73	597	37	8.2	0.51	41.1	6.2
Level of services							
Dispensaries	93	174	0	1.9 (2)^a^	0.0	27.9	0.0
Health centres	18	131	0	7.3 (4)^a^	0.0	38.4	0.0
First referral hospitals	15	479	38	31.9	2.53	20.3	7.9
Secondary referral hospitals	3	439	40	146.3	13.3	34.5	9.1
Urban-rural context							
Urban (2 districts)	20	639	54	16.0	1.35	31.5	8.5
Rural (4 districts)	109	584	24	1.3	0.06	25.0	4.1
Total	129	1223	78	9.5	0.60	27.2	6.1

^a ^Tanzanian national guidelines target

Table 2 shows that the required staffing levels at dispensaries are indeed available in Tanzania, with the number of qualified staff being only marginally less than that of the national standard, and those at the health centre level being nearly twice that of the national requirements. This is a surprising finding, since we expected a severe deficit of qualified personnel at these facility levels [20]. The importance of this finding is twofold. First, it provides optimism, considering the critical need for qualified personnel to achieve the MDGs in time. Second, however, it spurs a deeper investigation into the adequacy of national requirements, the distribution of personnel with respect to equity, the context within which they work, the use of the services by the expectant mothers and the role of qualified personnel in institutional quality.

### Describing the context

The selected study area represents four rural and two urban districts of Tanzania. The country has a reported maternal mortality rate (MMR) of 1100 deaths per 100 000 live births [21]. This places the country in a position of having among the six highest maternal mortality rates globally for the year 2000. The facilities in the study area are distributed between private for-profit, voluntary agencies and government services (Table 2). Most of the facilities are dispensaries (98), a few are secondary referral hospitals (3), and there are almost equal numbers of health centres and first referral hospitals (18 and 15). The number of facilities per district in rural districts is more than twice that of urban districts.

The majority of EmOC services are provided by voluntary agencies (private not-for-profit, often faith-based organizations) and government services. This is also reflected in the availability of the EmOC staff. The data show that voluntary agencies generally staff each facility with about twice as many qualified personnel as in government and private for-profit services (BemOC staff per facility are 14.0 to 8.2 and 4.5 for VA, G and PP, respectively, and CEmOC staff per facility are 0.87 to 0.51 and 0.45 for VA, G and PP, respectively). These figures confirm the findings of other audits in Tanzania [22].

The availability of qualified staff for each facility is much higher per district in urban areas compared to rural districts for both BEmOC (16.0 to 1.3) and CEmOC (1.35 to 0.06) staff. The same indicator shows that private for-profit facilities have a slightly higher availability per facility compared to voluntary agencies and government facilities (6.3% and 6.2%, respectively). There is little difference in the percentages of CEmOC personnel to the total EmOC personnel available at the facilities, except between the urban and rural districts (8.5% compared to 4.1%).

### Workload differences among qualified staff

Table 2 includes an overview of the workload differences among ownership and service levels. It should be noted that the data represent aggregate numbers at facility level and do not parse out details regarding the numbers of deliveries within facilities conducted by qualified or unqualified staff members. Table 2 shows that qualified government staff carry more than twice the burden of voluntary agency staff and more than six times the burden of private for-profit staff (41.1, 17.1 and 6.6, respectively). The corresponding figures across service levels and urban rural locations do not show very large differences, although the workload tends to be higher at the health centres and secondary referral levels, as well as in urban districts.

These findings demonstrate distinct location differences for HR, as well as differences in their workloads. It seems evident that private for-profit services bear smaller workloads, although the proportion of CEmOC to BEmOC staff is higher. This is not surprising, given their objectives of providing services to a smaller but wealthier segment of the population with higher expectations relative to available qualifications.

Furthermore, it seems clear that voluntary agencies staff each facility with a higher number of qualified personnel. We know that these services are often located in very remote rural areas, and it is somewhat surprising that they manage to recruit this level of qualified personnel to these areas. This could be due to factors such as better and more flexible HR policies, active training of local staff and employment contracts that include educational stipends. The fact that they have a reduced workload per qualified staff could be due to their remote location, but also due to the higher number of staff at each facility sharing the burden. Of significant importance is the flexibility with which each facility recruits personnel, based on the workload at the facility.

Government services differ from voluntary agencies as government facilities cannot easily recruit more personnel as the workload increases, and they are also subject to less personnel redistribution flexibility. The high workload per qualified staff ratio at government facilities could be due to the relatively low number of staff per facility, but also to the large number of deliveries conducted at government services (secondary referral hospitals) in urban areas

The higher workload encountered at the health centres could be explained by the relatively high number of qualified staff at health centres compared to dispensaries, assuming mothers prefer to deliver at a facility with a higher number of qualified staff [23]. This issue will be discussed later in the article.

### Differences in the distribution of qualified staff

One can analyse differences in qualified staff distributions by examining their service levels, populations at the district level and urban and rural characteristics of the district.

There are large variations in availability of qualified BEmOC and CEmOC staff in the different health service levels across the districts. This difference is notable at dispensary and first referral hospital levels. As shown in Table 3, the dispensary level figures range from 5.0 to 1.1 for qualified BEmOC staff per facility. The variations at the health centre level are much smaller. Compared to minimum national standards (as described earlier), almost all the rural districts are understaffed at the dispensary level, while at health centre level this is true for the facilities in only one rural district.

**Table 3:** Distribution of qualified human resources across districts according to population and health service levels

	**Average qualified BEmOC staff per dispensary**	**Average qualified BemOC staff per health centre**	**Average qualified BemOC staff per first referral hospital**	**Average qualified CemOC staff per first referral hospital**	**BemOC qualified personnel per 100 000**	**CemOC qualified personnel per 100 000**
Moshi	5.0	8.5	8.5	1.5	257	18
Hai	2.3	7.5	38.5	3.5	78	3
Arusha	3.0	9.3	18.8	1.8	107	11
Arumeru	1.7	3.0	36.7	3.0	33	2
Hanang	1.3	7.3	^a^	0.0	21	0
Mbulu	1.1	4.7	81.0	4.0	88	4
Urban	4.0	8.9	13.7	1.7	159.6	13.5
Rural	1.6	5.6	39.0	2.6	51.3	2.1
Total	1.8	7.3	31.9	2.5	79.5	5.1

^a^Hanang district did not have a first referral hospital.

In terms of available qualified staff per population, Table 3 shows a greater than tenfold difference in the availability of BEmOC qualified staff across the districts (21/100 000 to 257/100 000). The relative difference is even higher in terms of CEmOC qualified staff, although comparisons across districts is difficult, given the presence of secondary referral hospitals in the urban districts. Nevertheless, only one rural district does not comply with national guidelines in terms of available BEmOC personnel per 100 000 residents.

Relating the distribution of HR to their EmOC quality does not allow a direct comparison with other studies because of different methods used. Nonetheless, it is useful to compare the availability of BEmOC and CEmOC staff to the availability of nurses and doctors, respectively. This comparison reveals that the staff availability described for the entire study area (5.1 CEmOC staff per 100 000 and 79.5 BEmOC staff per 100 000) is very similar to the previous WHO estimates in Tanzania (4.1 doctors per 100 000 and 85.2 nurses per 100 000), and that Tanzania has the lowest qualified staff availability compared to other African countries [24], as shown in Table 4. Thus, it is reasonable to assume the national guidelines probably accept an unreasonably low number of qualified personnel per facility and thus per population, compared to other countries with similar HR characteristics.

**Table 4:** Estimates of health personnel per 100 000 population in selected African countries

**Country**	**Year**	**Physicians**	**Nurses**
Angola	1997	7.7	114.5
Botswana	1994	23.8	219.1
Democratic Republic of Congo	1996	6.9	44.2
Ghana	1996	6.2	72.0
Lesotho	1995	5.4	60.1
Kenya	1995	13.2	90.1
Namibia	1997	29.5	168.0
South Africa	1996	56.3	471.8
Swaziland	1996	15.1	...
Tanzania	1995	4.1	85.2
Zambia	1995	6.9	113.1
Zimbabwe	1995	13.9	128.7

Source: WHO estimates of health personnel; 1998

Interestingly, Table 3 does not confirm the expected finding of a uniformly higher qualified staff availability in urban districts. On the contrary, the availability of qualified staff is higher in the rural areas in the first referral hospitals of both staff categories. The higher staffing levels at voluntary agency facilities, as shown in Table 2, explain this discrepancy.

It has already been demonstrated in previous articles that the rural areas in Northern Tanzania depend heavily on voluntary agency EmOC services [14,15]. These findings are interesting for two reasons. First, it is clear that the total availability of qualified HR is much lower in rural districts than in urban districts. Secondly, the available qualified rural district staff are located at higher levels of the health care pyramid. This observation might be perfectly logical given the low level of available resources while at the same time the service providers are striving for adequate quality services.

### Availability of human resources

It is also useful to examine whether there exists a relationship between qualified staff levels and health care distribution equity. Health care equity is assessed in terms of service use. First, there is the question of whether a relationship between expected workload and actual workload exists. Second is the question of whether an increased number of qualified staff translates into a higher use of health care services measured with EmOC indicators, and finally if a higher availability of qualified staff translates into an increased number of qualified EmOC facilities, both at facility and district level.

#### Expected and actual workload at district level

Using census data, we anticipate that an increased number of expected deliveries should be followed by an increased allocation of qualified HR. The figures, however, show that urban districts exhibit consistently higher numbers of qualified staff. Table 5 summarizes the large variations in expected workloads among the districts, ranging from 13 to 212 anticipated deliveries per qualified BEmOC. We observed the same variations in expected deliveries per qualified CEmOC personnel, ranging from 192 to 2302. Both variables indicate a higher expected workload per qualified staff member in rural districts compared to those in urban districts (85 per BEmOC, and 2063 per CEmOC in rural districts; 22 per BEmOC and 255 per CEmOC in urban districts). This discrepancy is likely explained by the fact that EmOC staff members at the secondary referral hospitals are included in these figures. By definition, this staff category is allocated not only to the district within which they are placed, but also to the entire study area.

**Table 5:** Expected and actual workload per qualified personnel in terms of deliveries, complications and Caesarean Sections across districts

	**Expected deliveries per total qualified BEmOC personnel**	**Deliveries per qualified BemOC personnel**	**Expected deliveries per total qualified CemOC personnel**	**Deliveries per qualified CemOC personnel**	**Complications per qualified BemOC personnel**	**Complications per qualified CemOC personnel**	**Caesarean sections per qualified CemOC personnel**
Moshi	13	18	192	252	6.4	92	40
Hai	56	21	1564	591	3.2	91	11
Arusha	32	50	309	478	11.9	115	46
Arumeru	131	26	2302	450	3.6	62	17
Hanang	212	34	^a^	^a^	1.5	^a^	^a^
Mbulu	50	26	1197	635	4.2	101	41
Urban	22	32	255	373	8.8	105	44
Rural	85	25	2063	609	3.5	86	23
Total	52	28	817	446	6.3	99	37

^a^Hanang district had no qualified CemOC facilities or CemOC staff

We might expect a similarly large variation in workload per qualified EmOC personnel, given the large variation in staff allocation, as outlined above. No such variation exists, however. While the anticipated workload of qualified BEmOC staff is nearly 400% in rural districts compared to urban districts, the actual workload in rural districts is only 80% of that in urban districts.

The same is found for CEmOC qualified personnel. The anticipated workload of qualified CEmOC staff is more than 800% higher in rural districts compared to urban, while the actual workload is only about 160% higher. Again, this shows that either there is a very low percentage of facility deliveries, or the mothers travel across district boundaries to urban districts for obstetric health care. Although the answer is likely complex, the figures support the latter: that the actual workload in urban districts is higher than the expected workload (32 actual to 22 expected for BEmOC personnel and 373 actual to 255 expected for CEmOC personnel). These excess obstetric deliveries likely originate in neighboring rural districts.

A more detailed discussion about these usage patterns was presented previously [14,15]. The workload, in terms of deliveries per qualified personnel, is nevertheless still higher in rural districts. The other actual workload indicators (complications and caesarean sections per qualified personnel) are higher in the urban districts. This could indicate that the referral system is functional, such that complicated deliveries and caesarean sections are conducted in areas where available qualified personnel are located.

#### Availability of qualified HR and use

The Spearman's rank analysis shows a significant positive correlation between resource availability and use (met need r_s _= .943, percent deliveries in EmOC facilities r_s _= .829). The BEmOC per 500 000 statistic is used as the indicator of available HR, as BEmOC staff are present at all levels of the health care pyramid, while the CEmOC staff are only present at the top of the pyramid.

Table 6 illustrates the association between the availability of qualified HR and HR use and the availability of qualified HR and the number of qualified EmOC facilities. There is an increased likelihood that pregnant women choose facilities with qualified personnel. This is verified by publications on Northern Tanzanian clinical data showing that patients voluntarily bypass low-quality services in favour of high-quality services [23].

**Table 6:** Spearman's rank correlation analysis between the rankings of available qualified BemOC staff, met need and percent deliveries in EmOC facilities (as measures of utilization) and number of qualified BemOC and CemOC facilities (as measures of quality) in the districts

		**BEmOC qualified personnel per 500 000**
Met need	Correlation coefficient	.943(**)
	Sig. (2-tailed)	.005
Percent deliveries in EmOC facilities	Correlation coefficient	.829(*)
	Sig. (2-tailed)	.042
BEmOC facilities per 500 000	Correlation coefficient	.314
	Sig. (2-tailed)	.544
CEmOC facilities per 500 000	Correlation coefficient	.886(*)
	Sig. (2-tailed)	.019

* Correlation is significant at the 0.05 level. ** Correlation is significant at the 0.001 level.

### Availability of qualified HR and qualified EmOC facilities

Interestingly, there is no correlation (r_s _= 0.314) between the availability of qualified HR and the number of qualified BEmOC facilities. There is, however, a correlation (r_s _= .886) between the availability of qualified HR and the number of CEmOC-providing facilities. These data imply that the availability of qualified HR could translate into a higher number of qualified services provided at the higher levels of the health care pyramid, but not at the lower levels of the health care pyramid. A possible explanation for this finding could be the importance of increased training levels needed in the complex process of service provision.

The finding emphasizes the significance of maintaining a health policy focus on process and context parameters necessary for the translation of resources into quality services. These include important issues such as that health care providers must implement more effective management, motivation and policy relevance strategies and increase equipment and drug availability. In a health reform context, this analysis supports the claim that HR must be treated as more than merely a resource, and should be afforded a larger voice in policy formulation [25-29].

It is not possible, using only the Spearman's correlation test, to firmly conclude the direction of the direct correlation found between availability of qualified HR and use. It could be that increased availability leads to increased use, and that increased use leads to increased availability. We have, however, argued previously in this article that HR allocation is inflexible in Northern Tanzania. Increased use of health care services would inevitably necessitate increased HR availability, which would also require a flexible pool of qualified personnel within the target population. The direction of the correlation is most likely due to increased availability of qualified HR, leading to increased HR use.

Similarly, we could not definitively confirm the direction of the correlation between the availability of qualified HR and the number of higher level EmOC facilities. We argue that it is unlikely that the correlation is due to an increased number of facilities leading to higher availability of HR, for the same reasons as above. Rather, it is more likely that there is a correlation between an increased number of qualified HR leading to an increased number of qualified facilities.

In the Tanzanian setting, given the lack of a flexible qualified staff pool, the above described correlation most likely shows that a lower availability of qualified staff per district leads to lower numbers of qualified facilities. We argue this on the basis of the low overall availability of qualified staff not allowing adequate staffing of newly-developed health care facilities. It is more likely that as new facilities are built, HR are distributed more sparsely across these facilities, reducing the likelihood of each unit's becoming a qualified EmOC facility

#### Services of increased quality are needed before an increased quantity of services can ensue

Based on the results and discussion above, a priority should be to provide an increased number of qualified BEmOC staff to the understaffed districts in order to improve quality and use of existing facilities. Ideally, and importantly, increased numbers of qualified HR are urgently needed. Assuming that no more resources are made available, we need a redistribution of resources.

Although many commentators suggest a shift from higher to lower health care system levels, we believe our data suggest that present high level services are needed to provide the requisite comprehensive emergency obstetric care services and other referral services, train other health personnel, provide contingency services to Northern Tanzanian patients and respond to the demand, rights and trust of the population. Our data suggest that the HR available to these high level services are already at a minimum and should not be reduced.

Our analysis therefore suggests a shift of HR between and among health centre and dispensary levels in almost all districts as a measure to improve access to qualified HR. We should increase the availability of qualified staff at these facilities rather than maintain substandard quality. An example of how to implement this would be to reduce by half the number of available dispensaries and use the released resources to upgrade the remaining facilities to health centre levels. The location of these new health centres should be selected based on equity measures, such as geographical position, to maximize equity concerns.

This scenario should increase quality service availability at the expense of inadequate quality coverage. Such a situation can occur successfully if the managerial capacity and sustainable policy environment are present to translate these resources into good-quality services. Increasing the geographical range in which good services are provided necessitates improving patient access to these services. Thus, we must adopt and implement policies for improving transportation and communications infrastructure to facilitate remote access to health care services as well as to other types of important services.

Reducing the number of nonqualified facilities to the benefit of qualified facilities is useful for the supply of emergency obstetric care services. It remains to be investigated if it also applies for other services. It is likely that it at least applies to all services requiring higher levels of qualifications.

Applying this proposed solution to our study illustrates the potential of such a redistribution mechanism. There are 174 qualified BEmOC personnel distributed across 93 dispensaries. Further investigation is needed to find the adequate number of qualified staff needed to ensure a qualified facility. However, to provide the requisite four qualified personnel per health centre (as proposed by Tanzanian HR policies), these 174 health workers should be distributed across 43 health centres rather than the 93 dispensaries in the study area. If all these 43 new health centres provided high-quality services at the BEmOC level, according to the United Nations guidelines, the coverage of these services would be equivalent to 14 BEmOC facilities per 500 000 people, compared to the current 1.6 BEmOC facilities per 500 000 people reported in the study area (Table 1). The accepted minimum according to the United Nations guidelines is four BEmOC facilities per 500 000 people.

**Figure 1:**
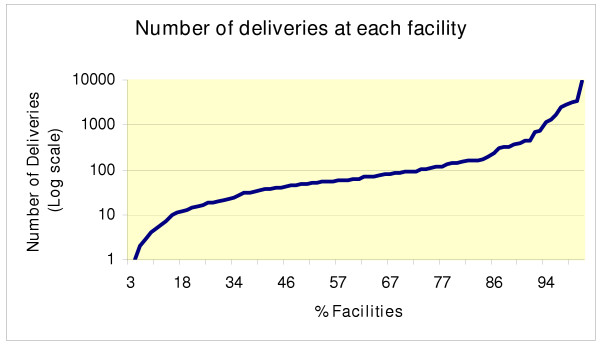
Distribution of deliveries per facility

Figure 1 illustrates the distribution of deliveries per facility on a log scale. More than 72% of the facilities conduct fewer than 100 deliveries per year. Almost all these facilities are dispensaries. Reducing the number of facilities would increase the number of deliveries per staff in these facilities, and thus also the quality of services. This example illustrates the potential of redistributing HR for quality rather than quantity care, at least for services requiring higher levels of qualifications, such as emergency obstetric care services.

Although this article demonstrates distribution differences in qualified HR across different contextual levels (urban/rural, ownership and health service level), it is useful to discuss briefly whether these differences are as inequitable as often described.

Agencies and commentators frequently criticize the relative, and thus perceived, excess HR working at higher levels of the health care pyramid. This excess, however, is understandable, given the extremely low number of qualified personnel in Tanzania. Indeed, Tanzanian national health authorities have few alternatives to placing qualified personnel in locations easily accessible by many people, and with adequate equipment and resources. It is better to provide adequate quality in higher-level facilities covering more people, than it is to provide inadequate quality in lower-level facilities covering fewer people, as argued previously in this article.

Neither should it be considered inequitable that much of the qualified HR are found in the private not-for-profit sector, given that this sector shares the same visions and objectives (equity, efficiency and quality) as the government. We should instead view this perspective in the wider context of the role of the state, in which the regulatory role is more important than that of the state as provider. Of concern is the total provision of good-quality services, accessible to all but not necessarily with the same overall coverage, given the severe resource constraints.

The issue at stake is not ownership or coverage, however, but rather health care quality, accessibility and trust. A high coverage of inadequate quality is not pro-poor. On the contrary, it has been demonstrated that low-quality services contribute to increased poverty [30]. The priority-setting issue with regard to HR in the study area should be to secure access to a determined threshold of quality, with increased availability of this level of quality provided as resources and managerial capacity are made available.

## Conclusion

We reveal in this article that there are adequate numbers of EmOC staff at the aggregated district level compared to Tanzanian HR requirements. However, there are large variations in the availability of qualified staff within these districts and severe understaffing at the dispensary levels in rural districts. The total number of available staff in Tanzania is also very low compared to other African countries. We also demonstrate that the availability of staff is concentrated within urban districts and in voluntary agency and government facilities.

Furthermore, there is a much higher workload per qualified staff at the government facilities. This is likely due to systematic understaffing at these facilities, given the expected workload. A significant reason for this may include the lack of flexibility in government facilities to adjust staffing levels to mirror the actual workload. The distribution of the qualified HR has important equity implications in that the pregnant women have to travel long distances to reach the qualified facilities.

Furthermore the article discusses possible relationships between higher availability of qualified staff, and the use and quality of services. The article argues that the availability of qualified staff is an important determinant of use of the services by the women, but that increased availability of qualified staff does not automatically translate into higher numbers of qualified facilities. We recommend that this relationship be investigated further, since developing more facilities necessitates greater management capability and enhanced clinical routines and motivation, as well as a critical exploration of the policy environment within which the services are provided.

The article encourages new HR targets for countries like Tanzania in which availability of quality services and not coverage alone should be the main focus. Increasing the availability of qualified HR must be a priority. Assuming however, that this increase will not happen in the near future, and given the present availability of resources at the different health care levels, we recommend a shift of resources within the dispensary level to reduce the total number of accessible dispensaries, rather than to redistribute HR from higher to lower quality levels. The remaining dispensaries should be upgraded to health centre-level quality, using the liberated resources, based on geographical and demographic parameters for equity maximization. The overall goal should be to improve access (transport and communications) to and coverage of quality services. Whether this is true for all types of services requires further investigation, but it is likely that it is relevant for all services requiring some form of higher qualifications.

The article also questions the criticism of uneven HR distribution between high and low levels of services often voiced, given the extremely low level of available resources. There are many good reasons for this distribution profile. The issue at stake is not ownership or coverage, but rather quality, accessibility, efficiency and trust of the services provided. The priority-setting discussions should therefore focus on defining an accepted threshold of quality under the present circumstances, determining how to distribute these services as widely as possible and deciding how to increase the availability of the current resources to optimize coverage of quality services. Service coverage indicators alone are not very useful for this process.

## List of abbreviations

BEmOC basic emergency obstetric care

CEmOC comprehensive emergency obstetric care

CBR crude birth rate

EmOC emergency obstetric care

G government services

HMIS health management information systems

HR human resources

MDGs Millennium Development Goals

PFP private, for-profit services

UNFPA United Nations Population Fund

UNICEF United Nations Children's Fund

VA voluntary agencies services

WHO World Health Organization

## Competing interests

The author(s) declare that they have no competing interests.

## Authors' contributions

ØEO was the principal investigator involved in all stages of the project as well as main author of the manuscript. SN participated in the conceptual outline of the project, methodological review and review of the manuscript. OFN supervised all stages of the project and participated in the analysis of the data and review of the manuscript. All authors read and approved the final manuscript.
